# Arbuscular mycorrhizal fungi enhance phosphate uptake and alter bacterial communities in maize rhizosphere soil

**DOI:** 10.3389/fpls.2023.1206870

**Published:** 2023-06-22

**Authors:** Yufan Lu, Yixiu Yan, Jie Qin, Luyan Ou, Xinyu Yang, Fang Liu, Yunjian Xu

**Affiliations:** ^1^ School of Agriculture, Yunnan University, Kunming, China; ^2^ Yunnan Key Laboratory of Plant Reproductive Adaptation and Evolutionary Ecology, Institute of Biodiversity, Yunnan University, Kunming, China; ^3^ School of Ecology and Environmental Science, Yunnan University, Kunming, Yunnan, China

**Keywords:** mycorrhizal symbiosis, nutrient mobilization, plant growth promotion, plant-microbe interactions, sulfur metabolism

## Abstract

Arbuscular mycorrhizal fungi (AMF) can symbiose with many plants and improve nutrient uptake for their host plant. Rhizosphere microorganisms have been pointed to play important roles in helping AMF to mobilize soil insoluble nutrients, especially phosphorus. Whether the change in phosphate transport under AMF colonization will affect rhizosphere microorganisms is still unknown. Here, we evaluated the links of interactions among AMF and the rhizosphere bacterial community of maize (*Zea mays* L.) by using a maize mycorrhizal defective mutant. Loss of mycorrhizal symbiosis function reduced the phosphorus concentration, biomass, and shoot length of maize colonized by AMF. Using 16S rRNA gene amplicon high-throughput sequencing, we found that the mutant material shifted the bacterial community in the rhizosphere under AMF colonization. Further functional prediction based on amplicon sequencing indicated that rhizosphere bacteria involved in sulfur reduction were recruited by the AMF colonized mutant but reduced in the AMF- colonized wild type. These bacteria harbored much abundance of sulfur metabolism-related genes and negatively correlated with biomass and phosphorus concentrations of maize. Collectively, this study shows that AMF symbiosis recruited rhizosphere bacterial communities to improve soil phosphate mobilization, which may also play a potential role in regulating sulfur uptake. This study provides a theoretical basis for improving crop adaptation to nutrient deficiency through soil microbial management practices.

## 1 Introduction

Phosphorus (P) is an essential macronutrient for plant growth and development (Wang et al., 2021b). Plants can only acquire free inorganic phosphate (Pi) from the soil, whereas Pi concentration and mobility in the soil are limited; thus, plants often suffer from Pi starvation (Du et al., 2020; Wang et al., 2021b). As important soil microorganisms, arbuscular mycorrhizal fungi (AMF) can symbiose with most plants and improve their host plants’ phosphorus status. When symbioses are formed, AMF provides Pi from soil to host plants through their hyphal networks (Smith and Read, 2008). However, the P in the soil mainly exists in organic matter and insoluble forms difficult for AMF to decompose directly (Tisserant et al., 2013; Zhang et al., 2022). AMF must depend on phosphate-solubilizing bacteria to complement their functional capabilities by mineralizing organic P resources (Falkowski et al., 2008; Zhang et al., 2022).

As an important mycorrhizal crop widely planted in the world, maize is sensitive to low Pi conditions (Liu et al., 2018; Ma et al., 2022). To improve the low Pi tolerance of maize, transgenic technology and the application of beneficial soil microbes (including AMF) have been used.

The rhizosphere inhabited by many microorganisms has been defined as the second plant genome (Kuzyakov and Razavi, 2019; Vetterlein et al., 2020). AMF symbiosis alters root exudates (Zhang et al., 2022; Xu et al., 2023a; Xu et al., 2023b), resulting in the rhizosphere microbiome being altered and stimulating soil nutrient turnover (Zhang et al., 2018a). AMF has been reported to influence the rhizosphere microbial composition involved in the decomposition of organic materials (Herman et al., 2012; Nuccio et al., 2013). Bacterial communities containing genes for alkaline phosphatases inhabiting on the AMF hyphal surface have been shown to enhance soil organic P mineralization (Zhang et al., 2018b). Jiang et al. (2021) show that AMF-carrying bacteria along their extraradical hyphae to organic P patches enhance organic P mineralization. Additionally, AMF also attracts some plant growth-promoting rhizobacteria that have potential functions in the mobilization of soil-insoluble nutrients, including P and sulfur (S) (Priyadharsini et al., 2016; Hestrin et al., 2019; Sun et al., 2020). There is an interaction between P and S absorption by plants (Mohamed et al., 2014; Sun et al., 2020). S starvation negatively impacts plant vitality when the P status is adequate (Sieh et al., 2013). Like P, inaccessible S in the soil also relies on interactions with AMF and associated microbes to promote their mobilization (Richardson et al., 2009). AMF has a crucial role in plant S metabolism *via* interaction with organo-sulfur mobilizing microbes (Narayan et al., 2022). Specific bacteria were attached to AMF symbiosis in agricultural soils, and some bacteria respond to the presence of specific AMF (Zhang et al., 2022), suggesting a high degree of specificity between bacteria and AMF. However, whether AMF recruits specific rhizobacteria to help maize cope with low Pi stress and whether these bacteria have an association with S metabolism is still largely unknown.

Increasingly, researchers believe that plant functional genes are important factors in regulating rhizosphere microbiota (Cordovez et al., 2019; Zhang et al., 2019). Plant functional genes can drive rhizosphere microbial community assembly by regulating root phenotypes, root exudates, and transporter activities (Zhalnina et al., 2018; Yu P. et al., 2021; Liu et al., 2022). Changes in the structure and function of rhizosphere microorganisms can also affect the expression of plant functional genes and take plants some growth advantages (Berendsen et al., 2018). Compared with NRT1.1B (encoding a nitrate transporter and sensor) mutant, there are more microorganisms involved in the nitrogen cycle in the rhizosphere of wild rice (Zhang et al., 2019). In another study, plants inoculated with synthetic microbial communities, nitrate transporter coding genes, and nitrate reductase coding genes are downregulated, whereas many phosphorus starvation genes, phosphate transport, and metabolism genes are activated by synthetic communities (SynComs) (Wang et al., 2021a). However, whether the mycorrhizal symbiosis-related genes in plants can regulate microbiota in the rhizospheres under AMF inoculation remains unknown. In this study, mycorrhizal defective mutant and wild-type (WT) maize plants were planted in soils inoculated with AMF. We examined the rhizosphere bacterial community by 16S rRNA amplicon sequencing and determined the soil bacteria changes affected by AMF symbiosis. This study provides a theoretical basis for improving crop fitness through rhizosphere microbial management practices.

## 2 Materials and methods

### 2.1 Greenhouse experiment

The wild type of maize B73 and the maize mycorrhizal defective mutant *Mut* were used in this study. Before planting, the seeds of maize were surface-sterilized with 75% ethyl alcohol and germinated for 2 days at 28°C after washing with ddH_2_O. The soil used in this study was superficial soil (0–20 cm depth) collected from a maize field in Kunming, Yunnan Province, China (24°23′N, 102°10E) after maize was harvested. The soil type is red loam. Soil physicochemical properties were as follows: pH, 7.01; total nitrogen, 0.42 g·kg^−1^; total phosphorus, 1.59 g·kg^−1^; available phosphorus, 9.8 mg kg^-1^; total potassium, 8.35 g·kg^−1^. Each pot (28 cm in height and 25 cm in diameter) contained 10 kg of soil. The experiments included AMF-inoculated and uninoculated treatments. The AMF inoculation was the mixed addition of *Diversispora epigaea*, *Claroideoglomus etunicatum*, *Claroideoglomus walkeri*, *Funneliformis mosseae*, and *Rhizophagus intraradices* inoculums. These inoculums were gifted by the National Engineering Lab of Crop Stress Resistance Breeding, Anhui Agricultural University, Anhui Province, China. There were 200 spores of each AMF species used to prepare the AMF mix, and a total of 1,000 AMF spores per pot were added. AMF uninoculated treatments were realized by applying benomyl. Benomyl has been shown to effectively reduce AM colonization with minimal direct effects on plants (Hetrick et al., 1990; Smith et al., 2000) and widely used in the investigation related to AM fungi (Wang et al., 2018; Li et al., 2019; Chen et al., 2020; Kang et al., 2020). Maize plants were grown at 28°C/25°C under a 16-h-day/8-h-night cycle for 8 weeks at a greenhouse of Yunnan University in China. The plants were watered twice a week. Five replicates of each treatment were set.

### 2.2 Plant analyses

Plant samples were harvested to measure dry weight, shoot length phosphorus concentration, and mycorrhizal colonization rate. Maize plants were dried at 105°C for 30 min and then dried at 75°C to a constant weight to measure dry weight. Dried leaves were ground and sieved (0.25 mm) to detect the phosphorus concentration as follows: samples were firstly digested in H_2_SO_4_ with H_2_O_2_ as an additive, and then the total phosphorus concentration was determined by molybdenum blue colorimetry (Deng et al., 2020). Trypan blue staining was used to detect the mycorrhizal colonization rate (Liu et al., 2018) as follows: fresh root segments were soaked in 10% (v/v) KOH solution for 15 min, acidified in 2% HCl for 5 min, and then stained with 0.05% trypan blue solution for 12 h.

### 2.3 Rhizosphere soil and 16S rRNA gene sequencing

Amplicon sequencing was conducted on a total of 12 samples, including three groups (wild type, wild type inoculated with AMF, *Mut* inoculated with AMF) and four biological replicates of each group. Rhizosphere soil was collected as previously described (McPherson et al., 2018). Briefly, roots with attached soil (rhizosphere soil) were transported to 30 ml phosphate-buffered saline buffer (pH 7.5). The mixture was centrifuged and passed through an aseptic nylon mesh strainer (100 μm). The filtered liquid was then centrifuged, and the rhizosphere soil was collected. The rhizosphere soil was stored at −80°C for total DNA extraction. According to the manufacturer’s instructions, the PowerSoil^®^ DNA isolation kit was used to extract the total DNA of rhizosphere soils. 16S rRNA gene sequencing was performed by the company Sangon Biotech (Shanghai, China) using an Illumina MiSeq platform. The V3–V4 regions of the bacterial 16S rRNA gene were amplified with universal primers 341F: CCTACGGGNGGCWGCAG and 805R: GACTACHVGGGTATCTAATCC (Defez et al., 2017) for bacterial identification. The 16S amplicon analysis was performed as Xu et al. described (Xu et al., 2023b). In brief, the DADA2 method for amplicon sequence variant (ASV) inference was used to process the 16S rRNA gene amplicon data; relative abundances of each taxon in a sample were calculated by proportional normalization of each sample by its sequencing depth. The random forest analysis was conducted by the “randomForest” R package as Xu et al. described (Xu et al., 2023b).

### 2.4 Data analysis

All the raw sequence data for the rhizosphere bacterial community have been deposited in the National Center for Biotechnology Information (NCBI) Sequence Read Archive under accession number PRJNA871781. Statistics on sequencing data were calculated using the R statistical programming software. Other statistical analyses were made using SPSS (SPSS26, Inc., Chicago, IL, USA). Statistical tests used were detailed in each figure legend. Redundancy analysis (RDA) was used to visualize and quantify the correlations. RDA was performed using the RDA command of the vegan package of R (Version 3.6.0) (R Core Team, 2019). The correlation heat maps were constructed by the corrplot package (Wei and Simko, 2021) and the ggcorrplot package (Tian et al., 2021) in R.

## 3 Results

### 3.1 Mycorrhizal defect hinders maize growth and phosphate uptake under AMF colonization

**Figure 1:**
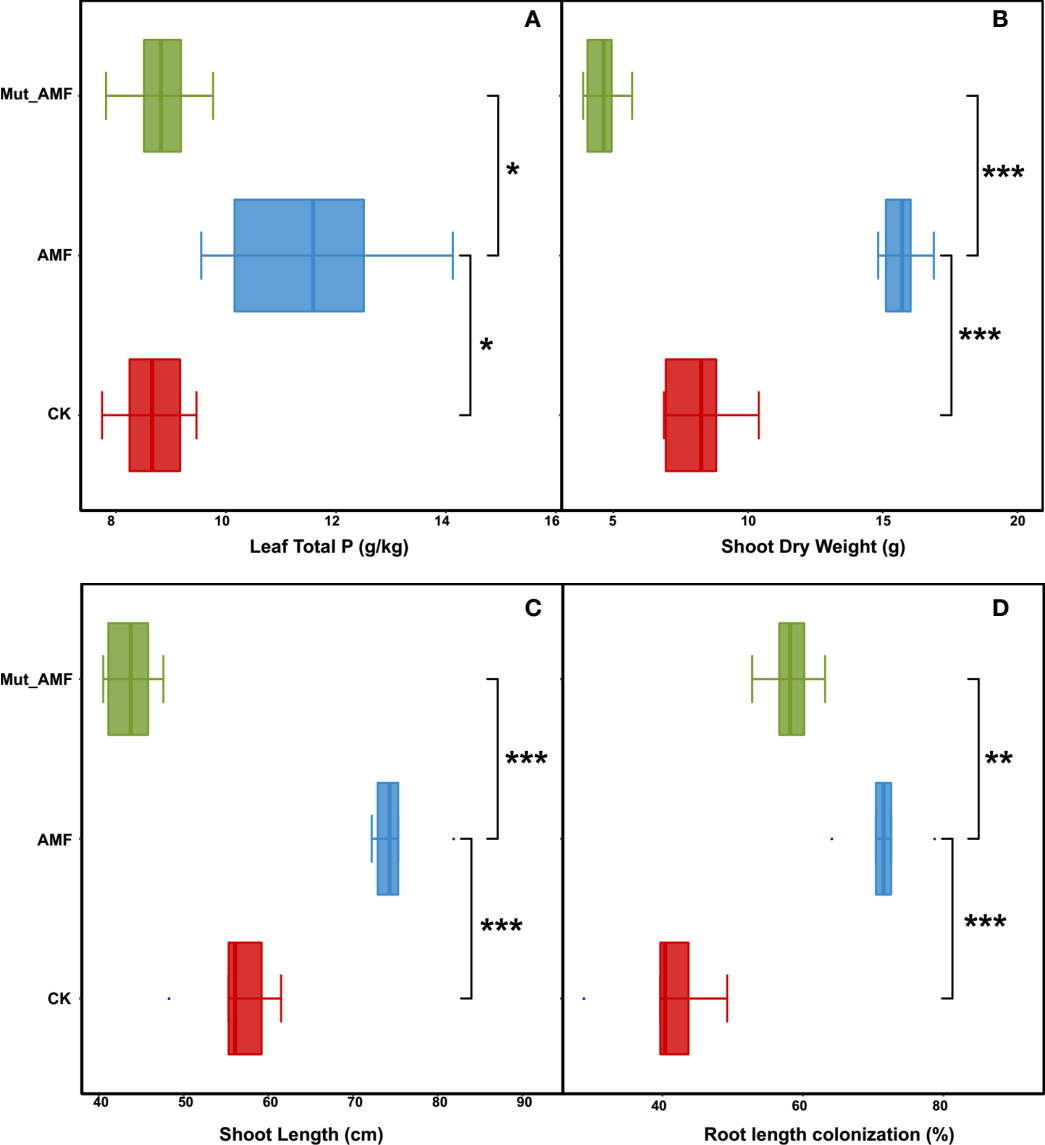
Arbuscular mycorrhizal fungal inoculation promotes maize growth and phosphate uptake. **(A)** Total P concentrations of plant. **(B)** Dry weight of shoot. **(C)** Shoot length. **(D)** Root length colonization rate. CK, wild-type maize without AMF (arbuscular mycorrhizal fungi) inoculation; AMF, wild-type maize inoculated with mix of *Diversispora epigaea*, *Claroideoglomus etunicatum*, *Claroideoglomus walkeri*, *Funneliformis mosseae*, and *Rhizophagus intraradices*. *Mut*, mycorrhizal defective mutant. The value of root length colonization rate was from three biological replicates with approximately 150 root segments per treatment. Bars are means ± SE and statistical differences were determined by two-sided t-test (***p < 0.001; **p < 0.01; *p < 0.05).

To investigate the effect of mycorrhizal defective mutant *Mut* on AM symbiosis and maize growth, the wild type and *Mut* mutant were inoculated with AMF. Eight weeks later, maize plants showed distinguishable phosphorus (P) content and phenotypes. AMF inoculation significantly increased the leaf P concentrations of wild-type maize plants by 33.86% and 31.39% compared with non-inoculated wild type (CK) and AMF-inoculated *Mut* mutant, respectively (**Figure 1A**). In addition, compared with CK, AMF inoculation significantly increased the shoot dry weight and shoot length of wild-type plants, but the *Mut* mutant reduced these performances of plants from AM symbiosis (**Figures 1B, C**). Moreover, the mycorrhizal colonization rate of AMF-inoculated wild-type maize was 71.48% (**Figure 1D**), whereas it was only 58.20% in AMF-inoculated *Mut*. Above all, AMF inoculation improved maize growth and promoted P absorption for maize, but the mycorrhizal defect reduced the accumulation of phosphorus, plant biomass, and shoot length of maize colonized by AMF.

### 3.2 AM symbiosis has a distinct rhizosphere bacterial community

The difference of bacterial community structures in the rhizosphere of CK, AMF-colonized wild type, and AMF-colonized *Mut* was confirmed by non-metric multidimensional (NMDS). NMDS analyses showed that bacterial community structures in the rhizosphere of wild type under AMF colonization were distinguishable from those without AMF colonization (stress value = 0.0846, **Figure S1A**), indicating a significant effect of AMF colonization on soil microbiome assembly. Additionally, significant differences were also observed between the rhizosphere bacterial structures of AMF colonized wild-type vs. AMF colonized *Mut* mutant (stress value = 0.0607, **Figure S1B**), suggesting that the *Mut* mutant affects microbiome assembly in the rhizosphere of maize under AMF colonization.

### 3.3 Rhizosphere bacteria act as biomarkers for AMF symbiosis

**Figure 2:**
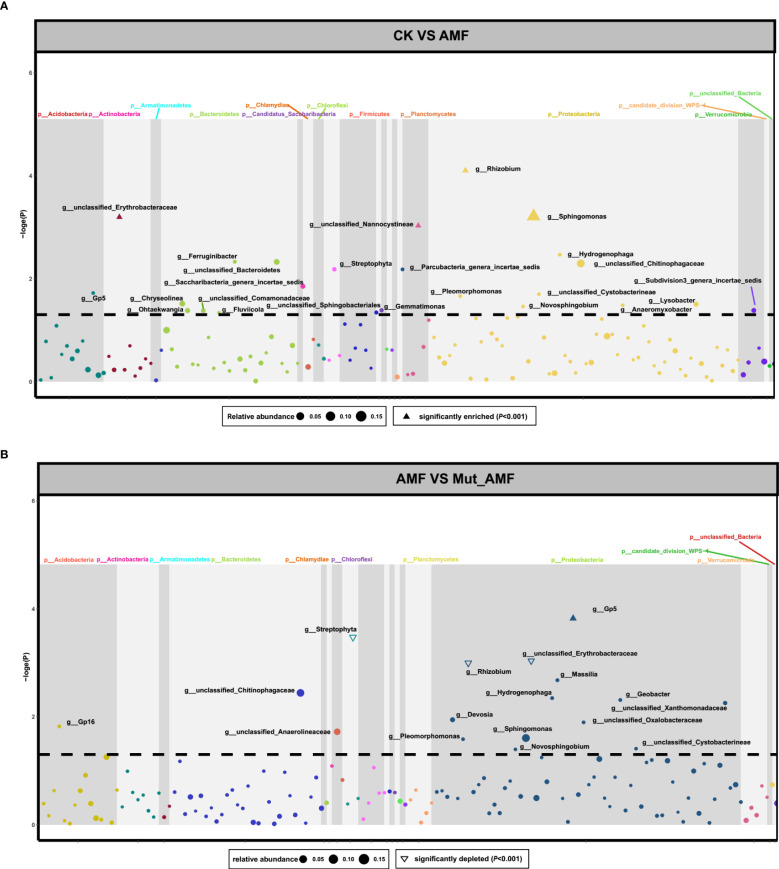
Changes of bacterial abundances in the rhizosphere of maize symbiosis with arbuscular mycorrhizal fungi. The dot represents the genera of bacteria. The Manhattan diagrams show changes of bacterial abundances in the rhizosphere of non-AMF (arbuscular mycorrhizal fungi) colonized (CK) vs. AMF colonized wild-type (AMF) **(A)**, and in the rhizosphere of AMF colonized wild-type vs. AMF colonized *Mut* mutant (Mut_AMF) **(B)**. The solid triangles represent significantly enriched bacteria. The hollow triangles represent significantly depleted bacteria, and the dot represents bacteria with no change. The dotted lines refer to the q value (-logP) of 0.05.

We further compared the relative abundance of rhizosphere bacteria between CK and AMF and between AMF-colonized wild-type and AMF-colonized *Mut*. Four genera of bacteria were enriched in the rhizosphere of maize colonized by AMF, namely, *Sphingomonas*, *Rhizobium*, *unclassified_Erythrobacteraceae*, and *unclassified_Nannocystineae* (**Figure 2A**). All four genera of bacteria belong to Proteobacteria. In the group of AMF-colonized wild type vs. AMF-colonized *Mut*, *Gp5* was significantly enriched, whereas *Streptophyta*, *Rhizobium*, and *unclassified_Erythrobacteraceae* were significantly depleted in the rhizosphere of AMF- colonized *Mut* (**Figure 2B**). *Gp5* belongs to Acidobacteria, and *Streptophyta* belongs to Cyanobacteria_Chloroplast. **Figure 2** reveals that *Rhizobium* and *unclassified_Erythrobacteraceae* were enriched in the rhizosphere of AMF- colonized wild type but depleted in *Mut*.

**Figure 3:**
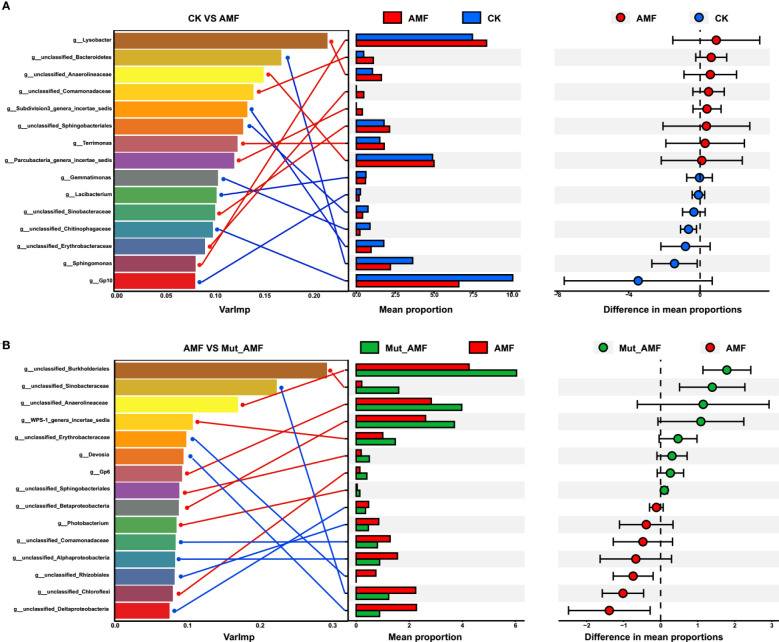
Analysis of biomarker bacterial genera by random forest modeling. The top 15 bacterial genera were identified by performing random forest classification of the relative abundance of the rhizosphere bacteria in non-AMF (arbuscular mycorrhizal fungi) colonized (CK) vs. AMF colonized wild-type (AMF) **(A)**, and in the rhizosphere of AMF colonized wild-type vs. AMF colonized *Mut* mutant (Mut_AMF) **(B)**. Biomarker taxa are ranked in descending order of importance to the accuracy of the model. Genus and its corresponding mean proportion were connected by lines.

We established a random forest to correlate AMF colonization with rhizosphere bacteria at the genus level. There were 15 genera identified as biomarker taxa in CK vs. AMF and AMF-colonized wild type vs. AMF-colonized *Mut*. Of these, eight genera showed higher relative abundance in AMF colonization compared to CK, namely, *Lysobacter*, *unclassified_Comamonadaceae*, *Terrimonas*, *unclassified_Anaerolineaceae*, *Parcubacteria_genera_incertae_sedis*, *unclassified_Sinobacteraceae*, *Sphingomonas*, and *unclassified_Erythrobacteraceae* (**Figure 3A**), whereas seven genera, namely, *unclassified_Bacteroidetes*, *Subdivision3_genera_incertae_sedis*, *unclassified_Sphingobacteriales*, *Gemmatimonas*, *Lacibacterium*, *unclassified_Chitinophagaceae*, and *Gp10*, were higher in CK. In AMF-colonized wild-type vs. AMF-colonized *Mut*, there were seven genera that were higher in wild type than *Mut*, namely, *unclassified_Sinobacteraceae*, *unclassified_Erythrobacteraceae*, *Devosia*, *unclassified_Comamonadaceae*, *unclassified_Alphaproteobacteria*, *unclassified_Rhizobiales*, and *unclassified_Deltaproteobacteria*, whereas eight genera with lower relative abundance were identified in AMF-colonized *Mut* compared with wild type (**Figure 3B**), namely, *unclassified_Burkholderiales*. *unclassified_Anaerolineaceae*, *WPS-1_genera_incertae_sedis*, *Photobacterium*, *unclassified_Betaproteobacteria*, *unclassified_Sphingobacteriales*, *Gp6*, and *unclassified_Chloroflexi*. Among these different bacteria members, the genera of *unclassified_Erythrobacteraceae* showed higher relative abundance in the rhizosphere with AMF colonization compared with CK, whereas it was reduced in AMF-colonized *Mut*. LDA analysis further indicated that the significantly different genera *unclassified_Erythrobacteraceae* can serve as biomarker genera to distinguish whether maize is inoculated with AMF (**Figure S2**).

### 3.4 Potential functions of the rhizosphere bacterial community

**Figure 4:**
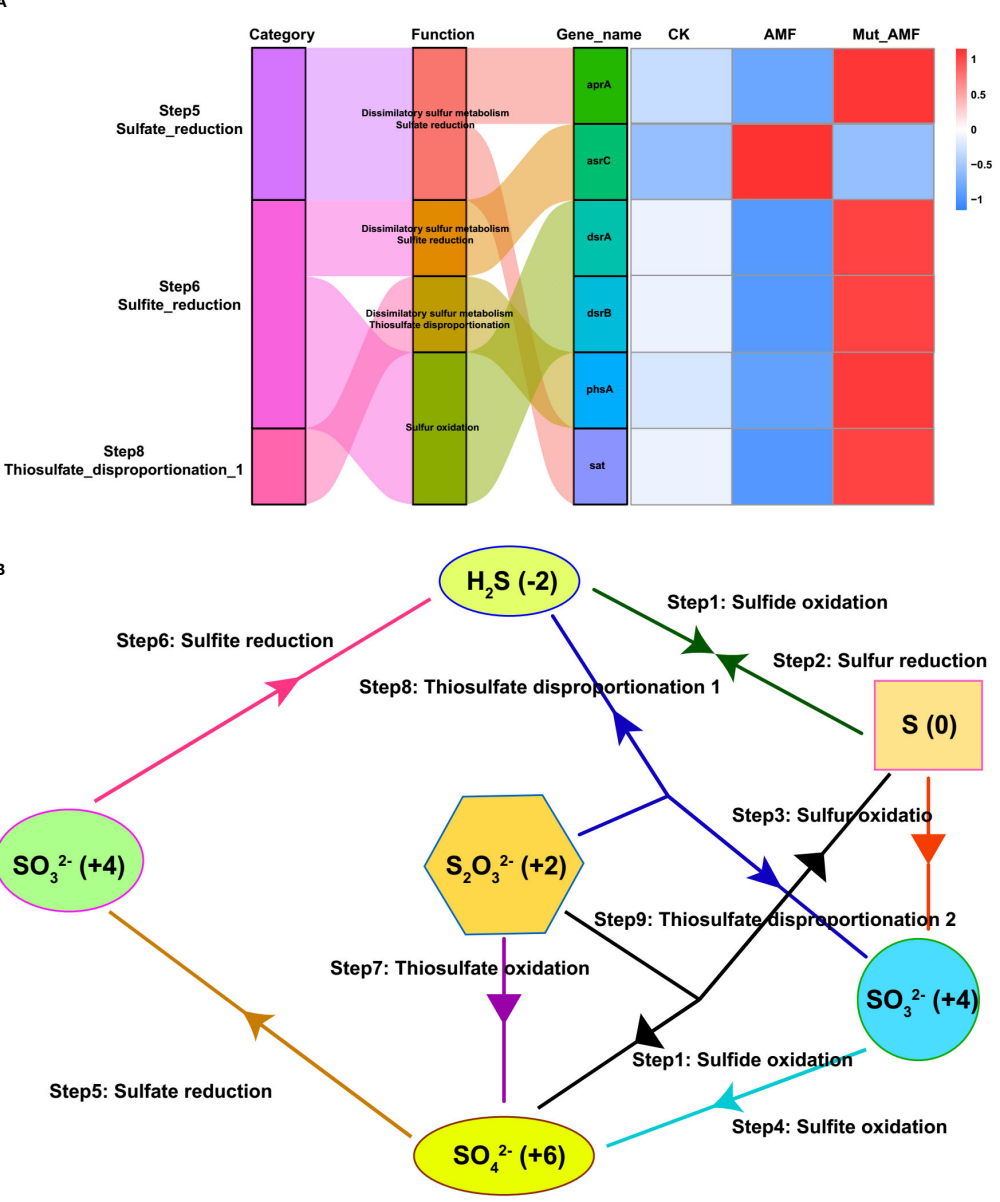
Sulfur metabolism-related genes in rhizosphere bacteria and their metabolic pathways. **(A)** Sankey diagram for sulfur metabolism pathway connected with related genes. The width of the flow is proportional to the number of bacterial genera. The right heatmap represents relative abundance of sulfur metabolism-related genes in the rhizosphere of non-AMF (arbuscular mycorrhizal fungi) colonized (CK), AMF colonized wild-type (AMF), and AMF colonized wild-type vs. AMF colonized *Mut* mutant (Mut_AMF). Relative abundance data were log2 transformed. **(B)** Sulfur metabolism.

The Functional Annotation of Procaryotic Taxa (FAPROTAX) analysis was further conducted to evaluate the different functions of bacterial communities in the rhizosphere of CK, AMF-colonized wild type, and AMF-colonized *Mut* mutant. Compared with CK and AMF-colonized *Mut* mutants, the rhizosphere of the AMF-colonized wild type had relatively higher bacterial abundance with 14 functions (**Figure S3**). Among the 14 functions, three sulfate (S) metabolism-related functions (respiration_of_sulfur_compounds and sulfate_respiration) were included. To explore the mechanism of rhizosphere bacteria associated with AMF inoculation in S metabolism, the Kyoto Encyclopedia of Genes and Genomes (KEGG) pathways analysis was conducted. Hereby, four pathways related to three steps of S metabolism (sulfate reduction, sulfite reduction, and thiosulfate disproportionation) were identified, namely, sulfate reduction, sulfite reduction, thiosulfate disproportionation, and sulfur oxidation (**Figures 4A, B**). Sulfate reduction, sulfite reduction, and thiosulfate disproportionation belong to dissimilatory sulfur metabolism. Evidence for dissimilatory sulfur metabolism and sulfur oxidation was sought by whether the bacteria containing genes necessary to perform sulfate reduction, sulfite reduction, and thiosulfate disproportionation, including sulfate adenylyltransferase gene (*sat*), adenylylsulfate reductase alpha gene (*aprA*), dissimilatory sulfite reductase alpha and beta subunits gene (*dsrAB*), and anaerobic sulfite reductase gene (*asrC*) (Cai et al., 2021; Kieft et al., 2021; Yu X. et al., 2021). The *sat* and *aprA* genes are involved in sulfate reduction, *phsA* is involved in thiosulfate disproportionation, *dsrAB* is involved in sulfite reduction, and *asrC* is involved in sulfite reduction. The rhizosphere soil from CK and AMF-colonized *Mut* mutant contained a higher relative abundance of *sat*, *aprA*, *phsA*, and *dsrAB*. Conversely, a lower relative abundance of *asrC* in the rhizosphere samples was detected in CK and AMF-colonized *Mut* mutant compared with the AMF-colonized wild type.

### 3.5 Correlation of rhizosphere bacteria related to sulfur metabolism with maize plant performances

**Figure 5:**
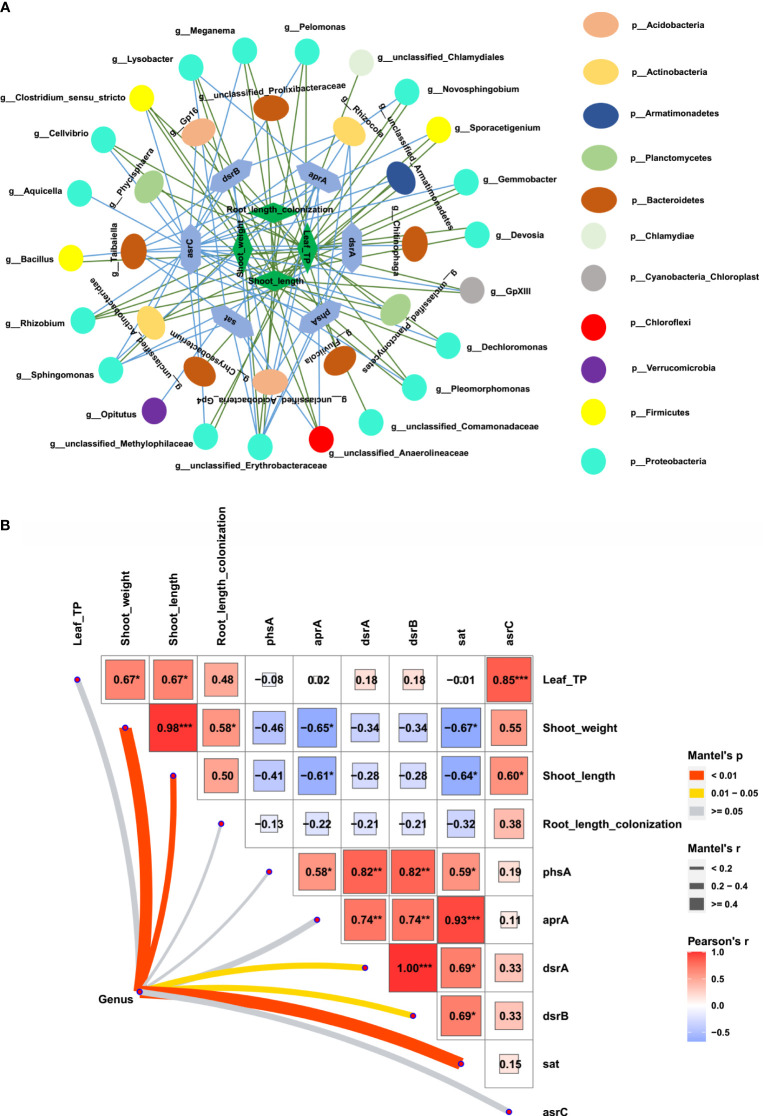
Correlation of sulfur metabolism- related rhizosphere bacterial genera and maize growth. **(A)** Network of sulfur metabolism- related rhizosphere bacterial genera, sulfur metabolism- related genes, and maize plant performances (shoot weight, shoot length, root length colonization, and shoot phosphorus concentration). **(B)** Correlation of sulfur metabolism- related rhizosphere bacterial genera with sulfur metabolism- related genes and maize performances.

To further explore whether the bacteria associated with S metabolism correlate with maize growth, we first identified the bacterial genera that contained at least one of *sat*, *aprA*, *phsA*, *dsrA*, *dsrB*, or *asrC*. A total of 59 genera related to S metabolism were identified. Among them, the relative abundance of 34 bacterial genera was increased in the rhizosphere of the AMF-colonized wild type but decreased in the AMF-colonized *Mut* mutant. A relationship network between plant performances and the 34 bacterial genera was constructed. Almost all the 34 genera have a positive relationship with shoot length, shoot weight, leaf P concentration, and root length colonization (**Figure 5A**). Furthermore, significant correlations among all the 59 bacterial genera, S metabolism genes, and plant growth parameters were summed up as a heatmap (**Figure 5B**). The six S metabolism-related genes (*sat*, *aprA*, *phsA*, *dsrA*, *dsrB*, and *asrC*) showed a significant positive correlation with each other. Both *aprA* and *sat* showed a significant negative correlation with shoot length and shoot weight of maize, whereas *phsA*, *dsrA*, *dsrB*, and *asrC* tended to show a negative correlation with shoot length, shoot weight, leaf P concentration, and root length colonization. Most of the 59 genera had a significant relationship with shoot weight and shoot length.

## 4 Discussion

Symbiosis with AMF is important for the growth and development of maize (Liu et al., 2018; Ma et al., 2022). The beneficial effects of AMF symbiosis are often related to improving the host plant to obtain nutrients from the soil, especially P (Felföldi et al., 2022). In our study, the contribution of *Mut* on P uptake through AMF symbiosis was confirmed. Statistically, the P concentration in maize plants was significantly increased post AMF inoculation, but it was reduced in the AMF- colonized *Mut* mutant compared with the wild type (**Figure 1**), suggesting that *Mut* plays roles in phosphate uptake for maize under AMF symbiosis.

Comparison between AMF- colonized and non-colonized rhizosphere samples (**Figure 2A**) indicated that AMF colonization affects the composition of the bacterial communities in the rhizosphere of maize. Emmett et al. also revealed a distinct bacterial community associated with *Glomus versiforme* by 16S rRNA gene sequencing (Emmett et al., 2021). However, the loss of the mycorrhizal symbiosis-related gene changed the bacterial community structure related to AMF symbiosis (**Figure 2B**). The changes of rhizosphere bacteria from the *Mut* mutant and wild type under AMF colonization showed that they had a preference for recruiting different soil bacterial communities. *Rhizobium* and *unclassified_Erythrobacteraceae* were depleted in the rhizosphere of AMF-colonized *Mut* but enriched in AMF-colonized wild type, which may be the specific microbial species greatly affected by the *Mut* with AM symbiosis. Rhizobia can symbiose with the roots of the host plant to improve host plant growth and yield (Yadav, 2021). Igiehon and Babalola (2021) showed that co-inoculation of soybean with rhizobium and AMF improved soybean yield under drought-stress conditions. The rhizobia has also been reported synergistically with AMF to promote root colonization, plant growth, and nitrogen acquisition (Liu et al., 2023; van der Heijden et al., 2023). The microbial species recruited in the mycorrhizal rhizosphere of maize were likely to help maize to obtain phosphate through the mycorrhizal pathway. AMF symbiosis changed exudates released into the rhizosphere soil to attract microorganisms, probably resulting in the directional enrichment of some special microorganisms (Jiang et al., 2021; Ma et al., 2022; Xu et al., 2023a; Xu et al., 2023b). The changes of rhizosphere bacteria in AMF-colonized *Mut* maize may result from the reduced AMF colonization rate, which might monitor changes of exudates in soil, and in turn, influence the recruitment of bacteria.

The changes of bacterial communities in the rhizosphere of the AMF-colonized *Mut* mutant suggested the key role of this maize gene in shaping soil bacterial communities under AMF colonization. Some plant genes have been revealed to affect rhizosphere microorganisms. For example, a rice nitrate transporter gene, NRT1.1B, has been reported to be associated with the recruitment of indica-enriched bacteria that could improve rice growth under organic nitrogen conditions using synthetic communities (Zhang et al., 2019); a salt-tolerant gene, *SST*, alleviates salt stress by regulating soil metabolites and microbiota in the rhizosphere of rice (Lian et al., 2020); and in soybean, transgenic *GsMYB10* shaped the rhizosphere microbial communities and harbored some beneficial microbes to resistant Al toxicity (Liu et al., 2023). In another way, the changes in soil microorganisms can also alter host gene expression (Stringlis et al., 2018; Rolfe et al., 2019). Rhizosphere microorganisms delay flowering by producing indole acetic acid (IAA) to downregulate genes that trigger flowering and stimulate further plant growth (Lu et al., 2018). The application of SynComs significantly upregulated many phosphate starvation responses (PSR) genes and downregulated nitrate transporter genes and nitrate reductase genes (Wang et al., 2021a). Therefore, it will be important to identify additional key genes in maize involved in AMF symbiosis or to screen the AMF-associated rhizosphere bacterial community as a potential molecular breeding target.

Cooperation between the host plant, AMF, and the soil bacteria is essential for plant growth in natural environments. Sulfur is an essential macro-element required for plant growth and is increasingly becoming limiting to crop yield (Narayan et al., 2022). Around 95% of sulfur is present in soil in organic form that cannot be directly utilized by plants, and plants rely on microorganisms in soil and rhizosphere to mobilize organic sulfur (Mahala et al., 2020). Many reports show that AMF symbiosis increases the sulfur content of plants (Narayan et al., 2022). For example, a study shows that AMF- colonized *Lolium perenne* increased cultivable sulfonate- mobilizing bacteria, helping in sulfur supply to the plant (Gahan and Schmalenberger, 2015). In this study, we found that AMF symbiosis reduced the proportion of rhizosphere bacteria related to sulfur reduction, including sulfate reduction, sulfite reduction, and thiosulfate disproportionation (**Figure 4**), which will increase inorganic sulfur content in the soil and promote plant to obtain sulfur. Dual inoculation with AMF and *Thiobacillus* has been revealed to improve the growth parameters and N, P, K, and S contents of onion and maize plants (Mohamed et al., 2014).

However, our study further found that bacteria associated with sulfur reduction were increased in the rhizosphere of *Mut* colonized by AMF (**Figure 4**), indicating that the sulfur reduction process is probably more efficient in AMF-colonized *Mut* root, which will reduce inorganic sulfur release from soil. There is an interaction between phosphorus and sulfur absorption by plants (Mohamed et al., 2014; Sun et al., 2020). AMF can affect the gene expression of plants in the process of sulfate transport, thus improving the nutritional status of plants. A sulfoquinovosyl diacylglycerol1 (SQD1) gene has been reported to affect Pi and S homeostasis (Sun et al., 2020). Mutation of *OsSQD1* increases the concentrations of sulfite but reduces the concentration of Pi in the Pi deficiency condition. The relative expression levels of Pi transporters *OsPT2*, *4*, *6*, and *8* were significantly lower in the *ossqd1* mutants. Another study has revealed that transcription factor *PHOSPHATE STARVATION RESPONSE 1* (*PHR1*, *At4g28610*), a key regulator of Pi starvation-responsive genes, also exerts a regulatory influence on the expression of sulfur transporter genes (*SULTR2.1* and *SULTR3.4*) and plays a key role in $\text{S}{\text{O}}_{4}^{2-}$ shoot-to-root flux during Pi deficiency (Rouached et al., 2011). Above all, genes associated with sulfur or phosphorus uptake are probably responsible for phosphorus and sulfur homeostasis at the same time. Since the *Mut* mutant negatively regulates maize P uptake (**Figure 1**), we speculated that *Mut* may have a potential role in affecting the transport of sulfur, and the changes in P uptake altered the rhizosphere microbiota, which may affect the sulfur uptake.

Above all, this study suggested that maize plants coordinate soil bacterial communities to mobilize phosphate and sulfur from the soil under AMF colonization to promote plant growth. The interaction between the AMF, plant gene, and rhizosphere bacteria could pave the way for technologies to modulate the rhizosphere bacteria to increase crop productivity and sustainability.

## Data availability statement

The datasets presented in this study can be found in online repositories. The names of the repository/repositories and accession number(s) can be found below: https://www.ncbi.nlm.nih.gov/,PRJNA871781.

## Author contributions

FL and YX contributed to the study conception and design. Material preparation and data collection were performed by YL and YY. Data analysis was performed by YL, YY, JQ, LO and XY. FL, YX, YL, and YY discussed and wrote the manuscript. All authors contributed to the article and approved the submitted version.

## Supplementary material

Supplementary Figure 1 Non-metric Multidimensional Scaling plot (NMDS) showing beta diversity of rhizosphere bacterial communities in non-AMF (arbuscular mycorrhizal fungi) colonized (CK) wild-type vs. AMF colonized wild-type (AMF) **(A)**, and in AMF colonized wild-type vs. AMF colonized *Mut* mutant (Mut_AMF) **(B)**.

Supplementary Figure 2 Linear discriminant analysis Effect Size (LEfSe) identified the differentially abundant bacteria in the rhizosphere of non-AMF (arbuscular mycorrhizal fungi) colonized (CK) wild-type vs. AMF colonized wild-type (AMF) **(A)**, and AMF colonized wild-type vs. AMF colonized *Mut* mutant (Mut_AMF) **(B)**.

Supplementary Figure 3 Functional Annotation of Procaryotic Taxa (FAPROTAX) analysis showing functional prediction of bacteria in the rhizosphere of non-AMF (arbuscular mycorrhizal fungi) colonized (CK) wild-type vs. AMF colonized wild-type (AMF) **(A)**, and AMF colonized wild-type vs. AMF colonized *Mut* mutant (Mut_AMF) **(B)**.

## References

[B1] Berendsen R. L. Vismans G. Yu K. Song Y. de Jonge R. Burgman W. P.. (2018). Disease-induced assemblage of a plant-beneficial bacterial consortium. ISME J. 12, 1496–1507. doi: 10.1038/s41396-018-0093-1 29520025 PMC5956071

[B2] Cai M. H. Luo G. Li J. Li W. T. Li Y. Li A. M. (2021). Substrate competition and microbial function in sulfate-reducing internal circulation anaerobic reactor in the presence of nitrate. Chemosphere 280, 130937. doi: 10.1016/j.chemosphere.2021.130937 34162109

[B3] Chen E. Liao H. Chen B. Peng S. (2020). Arbuscular mycorrhizal fungi are a double-edged sword in plant invasion controlled by phosphorus concentration. New Phytol. 226, 295–300. doi: 10.1111/nph.16359 31808168

[B4] Cordovez V. Dini-Andreote F. Carrión V. J. Raaijmakers J. M. (2019). Ecology and evolution of plant microbiomes. Annu. Rev. Microbiol. 73, 69–88. doi: 10.1146/annurev-micro-090817-062524 31091418

[B5] Defez R. Andreozzi A. Bianco C. (2017). The overproduction of indole-3-Acetic acid (IAA) in endophytes upregulates nitrogen fixation in both bacterial cultures and inoculated rice plants. Microb. Ecol. 74, 441–452. doi: 10.1007/s00248-017-0948-4 28197647

[B6] Deng S. Lu L. Li J. Du Z. Liu T. Li W.. (2020). Purple acid phosphatase 10c encodes a major acid phosphatase that regulates plant growth under phosphate-deficient conditions in rice. J. Exp. Bot. 71, 4321–4332. doi: 10.1093/jxb/eraa179 32270183 PMC7475256

[B7] Du E. Terrer C. Pellegrini A. F. A. Ahlström A. van Lissa C. J. Zhao X.. (2020). Global patterns of terrestrial nitrogen and phosphorus limitation. Nat. Geosci 13, 221–226. doi: 10.1038/s41561-019-0530-4

[B8] Emmett B. D. Lévesque-Tremblay V. Harrison M. J. (2021). Conserved and reproducible bacterial communities associate with extraradical hyphae of arbuscular mycorrhizal fungi. ISME J. 15, 2276–2288. doi: 10.1038/s41396-021-00920-2 33649552 PMC8319317

[B9] Falkowski P. G. Fenchel T. Delong E. F. (2008). The microbial engines that drive earth's biogeochemical cycles. Science 320, 1034–1039. doi: 10.1126/science.1153213 18497287

[B10] Felföldi Z. Vidican R. Stoian V. Roman I. A. Sestras A. F. Rusu T.. (2022). Arbuscular mycorrhizal fungi and fertilization influence yield, growth and root colonization of different tomato genotype. Plants 11, 1743. doi: 10.3390/plants11131743 35807693 PMC9269228

[B11] Gahan J. Schmalenberger A. (2015). Arbuscular mycorrhizal hyphae in grassland select for a diverse and abundant hyphospheric bacterial community involved in sulfonate desulfurization. Appl. Soil Ecol. 89, 113–121. doi: 10.1016/j.apsoil.2014.12.008

[B12] Herman D. J. Firestone M. K. Nuccio E. Hodge A. (2012). Interactions between an arbuscular mycorrhizal fungus and a soil microbial community mediating litter decomposition. FEMS Microbiol. Ecol. 80, 236–247. doi: 10.1111/j.1574-6941.2011.01292.x 22224699

[B13] Hestrin R. Hammer E. C. Mueller C. W. Lehmann J. (2019). Synergies between mycorrhizal fungi and soil microbial communities increase plant nitrogen acquisition. Commun. Biol. 2, 233. doi: 10.1038/s42003-019-0481-8 31263777 PMC6588552

[B14] Hetrick B. A. D. Wilson G. W. T. Todd T. C. (1990). Differential responses of C3 and C4 grasses to mycorrhizal symbiosis, phosphorus fertilization, and soil microorganisms. Can. J. Bot. 68, 461–467. doi: 10.1139/b90-061

[B15] Igiehon O. N. Babalola O. O. (2021). Rhizobium and mycorrhizal fungal species improved soybean yield under drought stress conditions. Curr. Microbiol. 78, 1615–1627. doi: 10.1007/s00284-021-02432-w 33686507 PMC7997835

[B16] Jiang F. Zhang L. Zhou J. George T. S. Feng G. (2021). Arbuscular mycorrhizal fungi enhance mineralisation of organic phosphorus by carrying bacteria along their extraradical hyphae. New Phytol. 230, 304–315. doi: 10.1111/nph.17081 33205416

[B17] Kang F. Yang B. Wujisiguleng Yang X. Wang L. Guo J.. (2020). Arbuscular mycorrhizal fungi alleviate the negative effect of nitrogen deposition on ecosystem functions in meadow grassland. Land Degrad Dev. 31, 748–759. doi: 10.1002/ldr.3491

[B18] Kieft K. Zhou Z. Anderson R. E. Buchan A. Campbell B. J. Hallam S. J.. (2021). Ecology of inorganic sulfur auxiliary metabolism in widespread bacteriophages. Nat. Commun. 12, 3503. doi: 10.1038/s41467-021-23698-5 34108477 PMC8190135

[B19] Kuzyakov Y. Razavi B. S. (2019). Rhizosphere size and shape: temporal dynamics and spatial stationarity. Soil Biol. Biochem. 135, 343–360. doi: 10.1016/j.soilbio.2019.05.011

[B20] Li J. Meng B. Chai H. Yang X. Song W. Li S.. (2019). Arbuscular mycorrhizal fungi alleviate drought stress in C3 (*Leymus chinensis*) and C4 (*Hemarthria altissima*) grasses *via* altering antioxidant enzyme activities and photosynthesis. Front. Plant Sci. 10, 499. doi: 10.3389/fpls.2019.00499 31114594 PMC6503820

[B21] Lian T. Huang Y. Xie X. Huo X. Shahid M. Q. Tian L.. (2020). Rice SST variation shapes the rhizosphere bacterial community, conferring tolerance to salt stress through regulating soil metabolites. mSystems 5, e00721-20. doi: 10.1128/mSystems.00721-20 33234605 PMC7687028

[B22] Liu L. Cheng L. Liu K. Yu T. Liu Q. Gong Z.. (2023). Transgenic soybean of *GsMYB10* shapes rhizosphere microbes to promote resistance to aluminum (Al) toxicity. J. Hazard Mater 455, 131621. doi: 10.1016/j.jhazmat.2023.131621 37187122

[B23] Liu Y. Evans S. E. Friesen M. L. Tiemann L. K. (2022). Root exudates shift how n mineralization and n fixation contribute to the plant-available n supply in low fertility soils. Soil Biol. Biochem. 165, 108541. doi: 10.1016/j.soilbio.2021.108541

[B24] Liu F. Xu Y. Han G. Wang W. Li X. Cheng B. (2018). Identification and functional characterization of a maize phosphate transporter induced by mycorrhiza formation. Plant Cell Physiol. 59, 1683–1694. doi: 10.1093/pcp/pcy094 29767790

[B25] Lu T. Ke M. Lavoie M. Jin Y. Fan X. Zhang Z.. (2018). Rhizosphere microorganisms can influence the timing of plant flowering. Microbiome 6, 231. doi: 10.1186/s40168-018-0615-0 30587246 PMC6307273

[B26] Ma J. Wang W. Yang J. Qin S. Yang Y. Sun C.. (2022). Mycorrhizal symbiosis promotes the nutrient content accumulation and affects the root exudates in maize. BMC Plant Biol. 22, 64. doi: 10.1186/s12870-021-03370-2 35123400 PMC8817564

[B27] Mahala D. M. Maheshwari H. S. Yadav R. K. Prabina B. J. Bharti A. Reddy K. K.. (2020). Microbial transformation of nutrients in soil: an overview. In Rhizosphere microbes: Microorganisms sustainability (Singapore) 23, 175–211. doi: 10.1007/978-981-15-9154-9_7

[B28] McPherson M. R. Wang P. Marsh E. L. Mitchell R. B. Schachtman D. P. (2018). Isolation and analysis of microbial communities in soil, rhizosphere, and roots in perennial grass experiments. J. Vis. Exp., 57932. doi: 10.3791/57932 30102263 PMC6126543

[B29] Mohamed A. A. Eweda W. E. E. Heggo A. M. Hassan E. A. (2014). Effect of dual inoculation with arbuscular mycorrhizal fungi and sulphur-oxidising bacteria on onion (*Allium cepa* l.) and maize (*Zea mays* l.) grown in sandy soil under green house conditions. Ann. Agric. Sci. 59, 109–118. doi: 10.1016/j.aoas.2014.06.015

[B30] Narayan O. P. Kumar P. Yadav B. Dua M. Johri A. K. (2022). Sulfur nutrition and its role in plant growth and development. Plant Signal Behav. doi: 10.1080/15592324.2022.2030082 PMC10730164 35129079

[B31] Nuccio E. E. Hodge A. Pett-Ridge J. Herman D. J. Weber P. K. Firestone M. K. (2013). An arbuscular mycorrhizal fungus significantly modifies the soil bacterial community and nitrogen cycling during litter decomposition. Environ. Microbiol. 15, 1870–1881. doi: 10.1111/1462-2920.12081 23360621

[B32] Priyadharsini P. Rojamala K. Ravi R. K. Muthuraja R. Nagaraj K. Muthukumar T. (2016). “Mycorrhizosphere: the extended rhizosphere and its significance,” in Plant-microbe interaction: an approach to sustainable agriculture. Eds. Choudhary D. Varma A. Tuteja N. (Singapore: Springer), 97–124.

[B33] R Core Team (2019). A language and environment for statistical computing (Vienna: R Foundation for Statistical Computing).

[B34] Richardson A. E. Barea J. M. Mcneill A. M. Prigent-Combaret C. (2009). Acquisition of phosphorus and nitrogen in the rhizosphere and plant growth promotion by microorganisms. Plant Soil 321, 305–339. doi: 10.1007/s11104-009-9895-2

[B35] Rolfe S. A. Griffiths J. Ton J. (2019). Crying out for help with root exudates: adaptive mechanisms by which stressed plants assemble health-promoting soil microbiomes. Curr. Opin. Microbiol. 49, 73–82. doi: 10.1016/j.mib.2019.10.003 31731229

[B36] Rouached H. Secco D. Arpat B. Poirier Y. (2011). The transcription factor PHR1 plays a key role in the regulation of sulfate shoot-to-root flux upon phosphate starvation in arabidopsis. BMC Plant Biol. 11, 19. doi: 10.1186/1471-2229-11-19 21261953 PMC3036608

[B37] Sieh D. Watanabe M. Devers E. A. Brueckner F. Hoefgen R. Krajinski F. (2013). The arbuscular mycorrhizal symbiosis influences sulfur starvation responses of *Medicago truncatula* . New Phytol. 197, 606–616. doi: 10.1111/nph.12034 23190168

[B38] Smith M. D. Hartnett D. C. Rice C. W. (2000). Effects of long-term fungicide applications on microbial properties in tallgrass prairie soil. Soil Biol. Biochem. 32, 935–946. doi: 10.1016/S0038-0717(99)00223-0

[B39] Smith S. E. Read D. J. (2008). Mycorrhizal symbiosis (London: Academic Press).

[B40] Stringlis I. A. Yu K. Feussner K. de Jonge R. Van Bentum S. Van Verk M. C.. (2018). MYB72-dependent coumarin exudation shapes root microbiome assembly to promote plant health. PNAS 115, E5213–E5222. doi: 10.1073/pnas.1722335115 29686086 PMC5984513

[B41] Sun Y. Jain A. Xue Y. Wang X. Zhao G. Liu L.. (2020). OsSQD1 at the crossroads of phosphate and sulfur metabolism affects plant morphology and lipid composition in response to phosphate deprivation. Plant Cell Environ. 43, 1669–1690. doi: 10.1111/pce.13764 32266981

[B42] Tian W. Xiang X. Wang H. (2021). Differential impacts of water table and temperature on bacterial communities in pore water from a subalpine peatland, central China. Front. Microbiol. 12. doi: 10.3389/fmicb.2021.649981 PMC8193233 34122363

[B43] Tisserant E. Malbreil M. Kuo A. Kohler A. Symeonidi A. Balestrini R.. (2013). Genome of an arbuscular mycorrhizal fungus provides insight into the oldest plant symbiosis. PNAS 110, 20117–20122. doi: 10.1073/pnas.1313452110 24277808 PMC3864322

[B44] van der Heijden M. G. de Bruin S. Luckerhoff L. van Logtestijn R. S. Schlaeppi K. (2016). A widespread plant-fungal-bacterial symbiosis promotes plant biodiversity, plant nutrition and seedling recruitment. ISME J. 10, 389–399. doi: 10.1007/s10725-023-00966-6 26172208 PMC4737930

[B45] Vetterlein D. Carminati A. Kögel-Knabner I. Bienert G. P. Smalla K. Oburger E.. (2020). Rhizosphere spatiotemporal organization–a key to rhizosphere functions. Front. Agron. 2. doi: 10.3389/fagro.2020.00008

[B46] Wang Y. Chen Y. F. Wu W. H. (2021b). Potassium and phosphorus transport and signaling in plants. J. Integr. Plant Biol. 63, 34–52. doi: 10.1111/jipb.13053 33325114

[B47] Wang C. Li Y. Li M. Zhang K. Ma W. Zheng L.. (2021a). Functional assembly of root-associated microbial consortia improves nutrient efficiency and yield in soybean. J. Integr. Plant Biol. 63, 1021–1035. doi: 10.1111/jipb.13073 33491865

[B48] Wang X. X. Wang X. Sun Y. Cheng Y. Liu S. Chen X.. (2018). Arbuscular mycorrhizal fungi negatively affect nitrogen acquisition and grain yield of maize in a n deficient soil. Front. Microbiol. 9. doi: 10.3389/fmicb.2018.00418 PMC5852317 29568292

[B49] Wei T. Simko V. (2021) R package ‘corrplot’: visualization of a correlation matrix. Available at: https://github.com/taiyun/corrplot.

[B50] Xu Y. Chen Z. Li X. Tan J. Liu F. Wu J. (2023a). The mechanism of promoting rhizosphere nutrient turnover for arbuscular mycorrhizal fungi attributes to recruited functional bacterial assembly. Mol. Ecol. 32, 2335–2350. doi: 10.1111/mec.16880 36762879

[B51] Xu Y. Chen Z. Li X. Tan J. Liu F. Wu J. (2023b). Mycorrhizal fungi alter root exudation to cultivate a beneficial microbiome for plant growth. Funct. Ecol. 37, 664–675. doi: 10.1111/1365-2435.14249

[B52] Yadav A. N. (2021). Beneficial plant-microbe interactions for agricultural sustainability. J. Appl. Biol. Biotech. 9, 1–4. doi: 10.7324/JABB.2021.91ed

[B53] Yu P. He X. Baer M. Beirinckx S. Tian T. Moya Y. A. T.. (2021). Plant flavones enrich rhizosphere oxalobacteraceae to improve maize performance under nitrogen deprivation. Nat. Plants 7, 481–499. doi: 10.1038/s41477-021-00897-y 33833418

[B54] Yu X. Zhou J. Song W. Xu M. He Q. Peng Y.. (2021). SCycDB: a curated functional gene database for metagenomic profiling of sulphur cycling pathways. Mol. Ecol. Resour. 21, 924–940. doi: 10.1111/1755-0998.13306

[B55] Zhalnina K. Louie K. B. Hao Z. Mansoori N. da Rocha U. N. Shi S.. (2018). Dynamic root exudate chemistry and microbial substrate preferences drive patterns in rhizosphere microbial community assembly. Nat. Microbiol. 3, 470–480. doi: 10.1038/s41564-018-0129-3 29556109

[B56] Zhang L. Feng G. Declerck S. (2018a). Signal beyond nutrient, fructose, exuded by an arbuscular mycorrhizal fungus triggers phytate mineralization by a phosphate solubilizing bacterium. ISME J. 12, 2339–2351. doi: 10.1038/s41396-018-0171-4 29899507 PMC6155042

[B57] Zhang J. Liu Y. X. Zhang N. Hu B. Jin T. Xu H.. (2019). NRT1.1B is associated with root microbiota composition and nitrogen use in field-grown rice. Nat. Biotechnol. 37, 676–684. doi: 10.1038/s41587-019-0104-4 31036930

[B58] Zhang L. Shi N. Fan J. Wang F. George T. S. Feng G. (2018b). Arbuscular mycorrhizal fungi stimulate organic phosphate mobilization associated with changing bacterial community structure under field conditions. Environ. Microbiol. 20, 2639–2651. doi: 10.1111/1462-2920.14289 29901256

[B59] Zhang L. Zhou J. George T. S. Limpens E. Feng G. (2022). Arbuscular mycorrhizal fungi conducting the hyphosphere bacterial orchestra. Trends Plant Sci. 27, 402–411. doi: 10.1016/j.tplants.2021.10.008 34782247

