# Supplementary figures and images for: Arbuscular mycorrhizal fungi enhance phosphate uptake and alter bacterial communities in maize rhizosphere soil
Source: Front Plant Sci. 2023 Jun 22;14:1206870. doi: 10.3389/fpls.2023.1206870 (PMC10325641; doi:10.3389/fpls.2023.1206870)

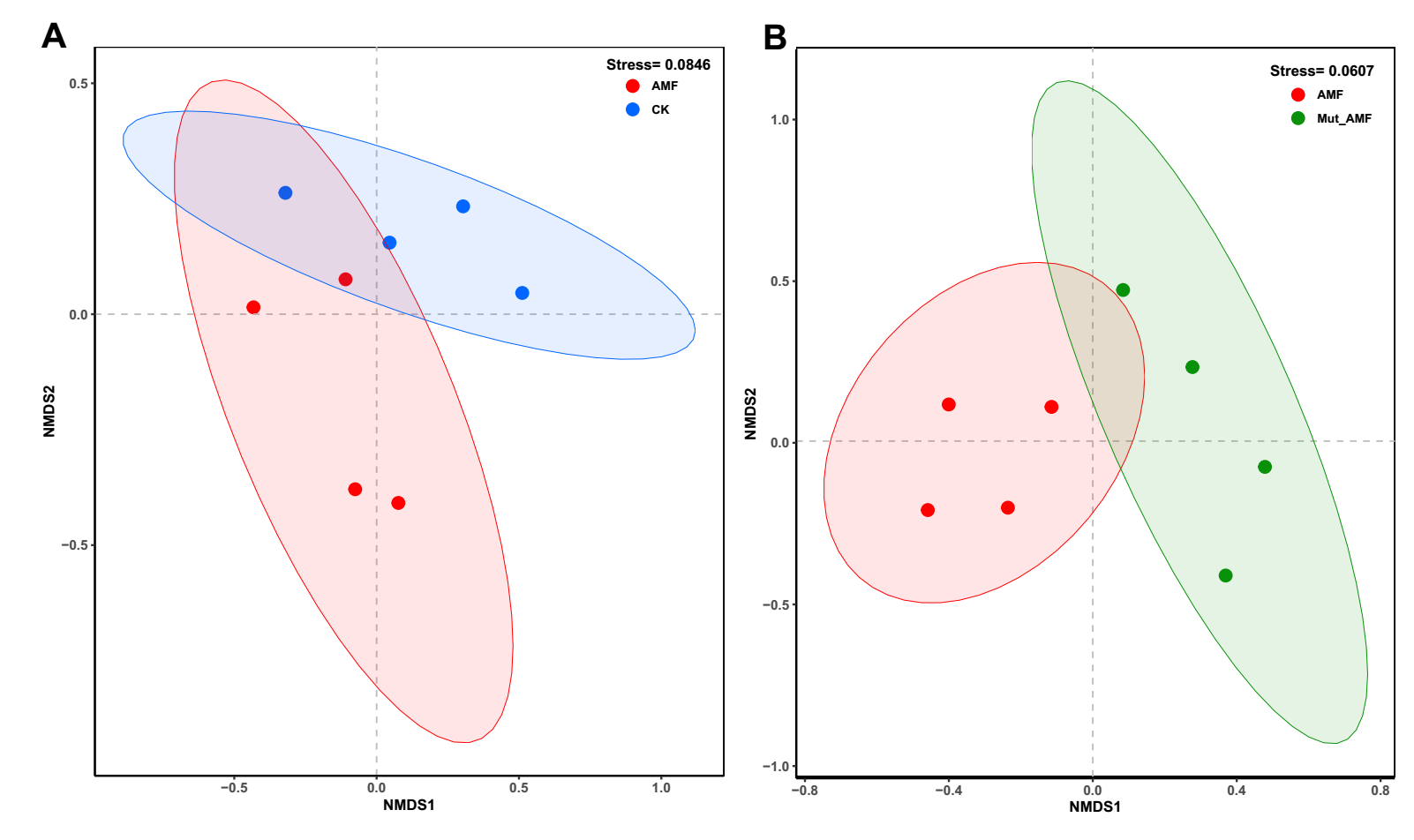

Supplement: Supplementary Figure 1 — Non-metric Multidimensional Scaling plot (NMDS) showing beta diversity of rhizosphere bacterial communities in non-AMF (arbuscular mycorrhizal fungi) colonized (CK) wild-type vs. AMF colonized wild-type (AMF) (A), and in AMF colonized wild-type vs. AMF colonized Mut mutant (Mut_AMF) (B). [file Image_1.tif]

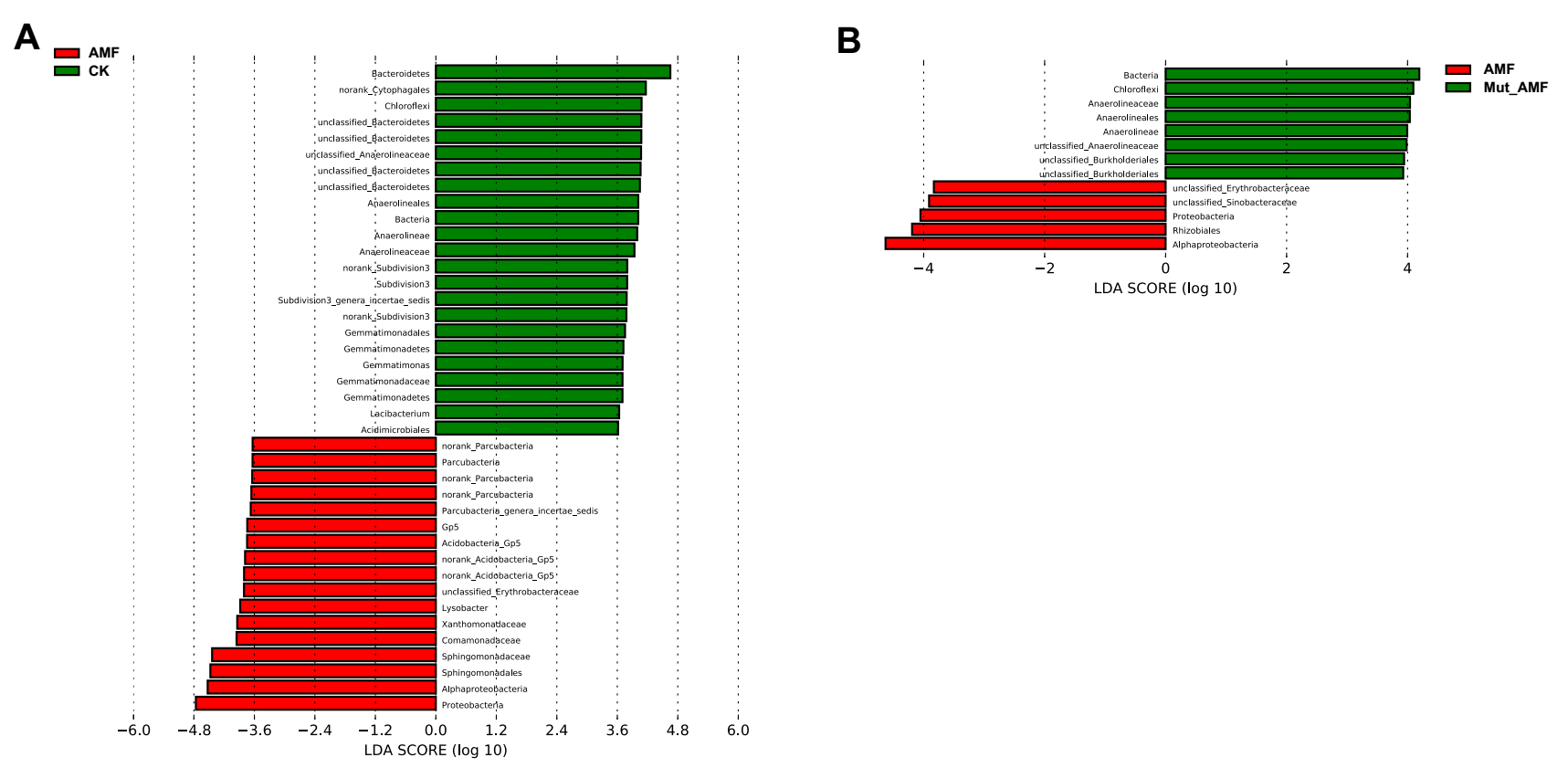

Supplement: Supplementary Figure 2 — Linear discriminant analysis Effect Size (LEfSe) identified the differentially abundant bacteria in the rhizosphere of non-AMF (arbuscular mycorrhizal fungi) colonized (CK) wild-type vs. AMF colonized wild-type (AMF) (A), and AMF colonized wild-type vs. AMF colonized Mut mutant (Mut_AMF) (B). [file Image_2.tif]

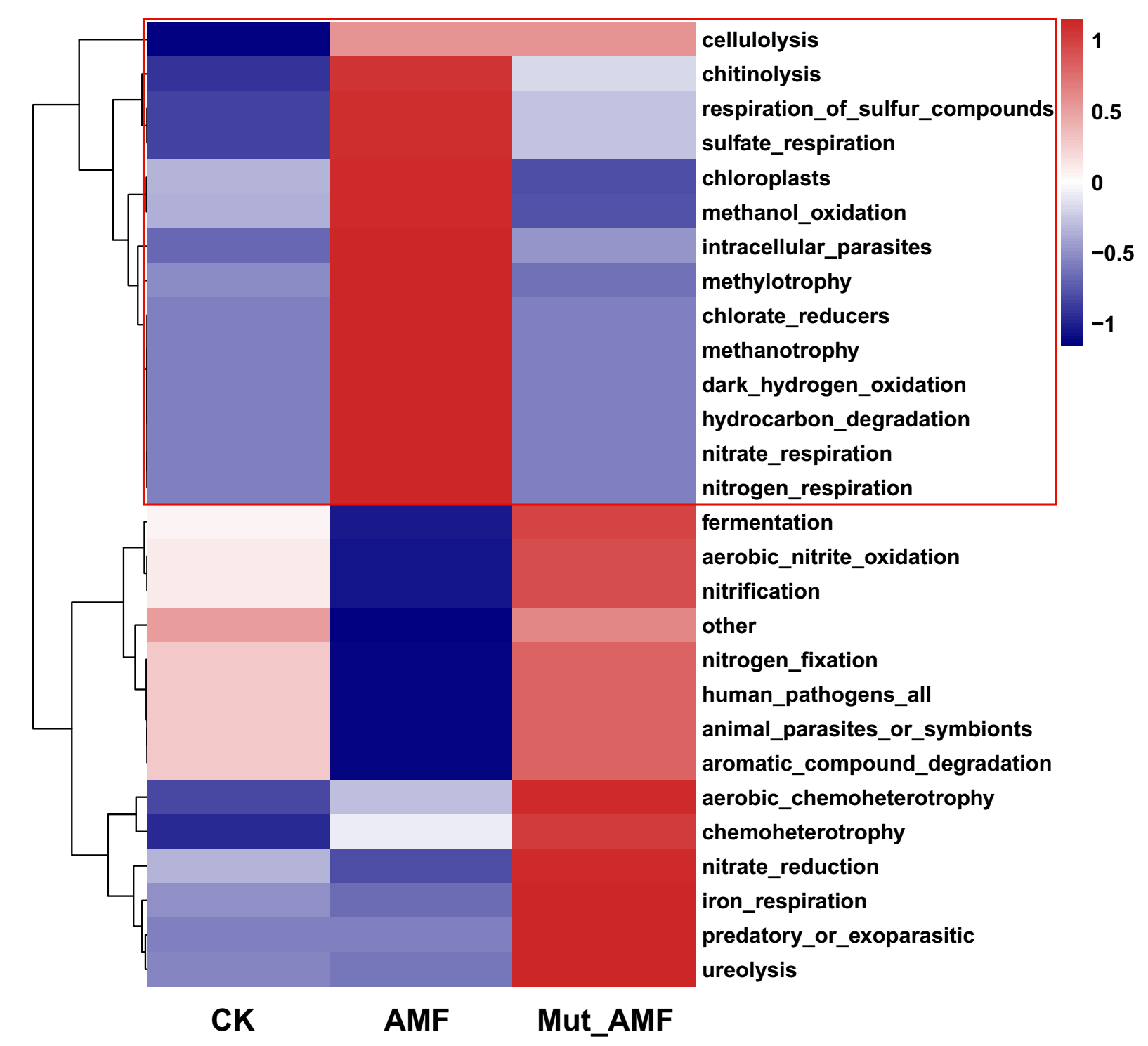

Supplement: Supplementary Figure 3 — Functional Annotation of Procaryotic Taxa (FAPROTAX) analysis showing functional prediction of bacteria in the rhizosphere of non-AMF (arbuscular mycorrhizal fungi) colonized (CK) wild-type vs. AMF colonized wild-type (AMF) (A), and AMF colonized wild-type vs. AMF colonized Mut mutant (Mut_AMF) (B). [file Image_3.tif]
